# Volatile Emission in Bracken Fern Is Induced by Jasmonates but Not by *Spodoptera littoralis* or *Strongylogaster multifasciata* Herbivory

**DOI:** 10.1371/journal.pone.0048050

**Published:** 2012-11-20

**Authors:** Venkatesan Radhika, Christian Kost, Gustavo Bonaventure, Anja David, Wilhelm Boland

**Affiliations:** 1 Department of Bioorganic Chemistry, Max Planck Institute for Chemical Ecology, Jena, Germany; 2 Plant Productivity System Research, Plant Science Center, RIKEN Yokohama Institute, Yokohama City, Japan; 3 Experimental Ecology and Evolution Research Group, Max-Planck Institute for Chemical Ecology, Jena, Germany; 4 Institute of Microbiology, Friedrich Schiller University Jena, Jena, Germany; 5 Department of Molecular Ecology, Max Planck Institute for Chemical Ecology, Jena, Germany; Odum School of Ecology, University of Georgia, United States of America

## Abstract

Jasmonate-mediated regulation of VOC emission has been extensively investigated in higher plants, however, only little is known about VOC production and its regulation in ferns. Here, we investigate whether the emission of VOCs from bracken fern *Pteridium aquilinum* is triggered by herbivory and if so - whether it is regulated by the octadecanoid signaling pathway. Interestingly, feeding of both generalist (*Spodoptera littoralis*) and specialist (*Strongylogaster multifasciata*) herbivores as well as application of singular and continuous mechanical wounding of fronds induced only very low levels of VOC emission. In contrast, treatment with jasmonic acid (JA) led to the emission of a blend of VOCs that was mainly comprised of terpenoids. Likewise, treatment with the JA precursor 12-oxo-phytodienoic acid (OPDA) and α-linolenic acid also induced VOC emission, albeit to a lower intesity than the JA treatment. Accumulation of endogenous JA was low in mechanically wounded fronds and these levels were unaffected by the application of oral secretions from both generalist or specialist herbivores. The emission of terpenoids upon JA treatment could be blocked with fosmidomycin and mevinolin, which are inhibitors of the MEP- and MVA pathways, respectively. These results indicate that similar to higher plants, terpenoid VOCs are produced via these pathways in bracken fern and that these pathways are JA-responsive. However, the very low amounts of terpenoids released after herbivory or mechanical damage are in stark contrast to what is known from higher plants. We speculate that *S. multifasciata* and *S. littoralis* feeding apparently did not induce the threshold levels of JA required for activating the MEP and MVA pathways and the subsequent volatile emission in bracken fern.

## Introduction

The emission of volatile organic compounds (VOCs) is a well-known indirect defence mechanism, by which plants recruit natural enemies (predators and parasitoids) of their herbivores [1], [2]. VOCs are generally believed to function as an ‘alarm signal’ that is generated by plants in distress. Depending on the type of stress (herbivore-/pathogen-attack or tissue damage) and the plant species involved, quantitatively and qualitatively different bouquets are released [3]. Terpenoids are the most abundant and structurally diverse class of VOCs that are released upon herbivore damage by many higher plants [4]. These can be a mixture of monoterpenes (C_10_), sesquiterpenes (C_15_), and homoterpenes (C_11_, C_16_), all of which are synthesized from a basic C_5_ unit, isopentenyl diphosphate (IPP) and dimethyallyl diphosphate (DMAPP) via either the cytosol-localized mevalonate (MVA) or plastid-localized methylerythritol (MEP) pathway [1], [4]. A huge body of literature is available on the regulation of VOC emission in many species of higher plant such as *Phaseolus lunatus*, *Zea mays* [5] *Gossypium hirsutum* [6], *Populus simoniix*, *Nicotiana attenuata, and Solanum tuberosum* [4], [7]. In contrast, very little is known about VOC emission in lower plants such as ferns (but see: [8], [9]).

In higher plants, octadecanoids are central regulators of defensive responses when the plant faces insect, mite, or fungal attackers [10]. The cascade, initiated by the mechanical wounding of cellular membranes [11], e.g. by caterpillar mandibles and their oral secretions, begins with the release of α-linolenic acid from cell membranes [1], [12], [13] and their subsequent conversion into 12-oxophytodienoic acid (OPDA). After further transformation to jasmonic acid and conjugation to L-isoleucine, the now-active hormone binds to a jasmonate ZIM domain (JAZ) protein [14], [15] and finally leads to local and systemic defensive responses, such as the emission of predator-attractive volatiles [8], [16], [17]. Both herbivory and continuous mechanical wounding [18] induce VOC emission via the octadecanoid pathway [1]. Interestingly, exogenous application of JA also triggers the emission of VOCs, rendering the use of this elicitor, a powerful methodological tool for the study of this indirect defence strategy. The qualitative and quantitative composition of the VOC blend emitted upon JA-treatment strongly resembles the one released after wounding or herbivory [5], [19]. Interestingly, significant qualitative differences in the VOC blend induced after application of early intermediates of the JA pathway namely 12-oxo-phytodienoic acid (OPDA) and α-linolenic acid have been reported, thereby providing insights into the underlying regulatory pathways that elicit the emission of volatile compounds [20]. In addition, the fungal peptide alamethicin (ALA) as a channel-forming elicitor of microbial origin is also known to induce VOC emission. Such channel-forming compounds are widely present in the oral secretions of many herbivorous insects [21]. Although VOC emission in higher plants has been intensively studied [7], comparatively very little is known about such defences in lower plants.

Ferns are the most ancient of extant plant groups with fossil records predating the early Devonian era (about 400 million years ago) [22]. They have been thriving on earth for about 200 million years before the first flowering plants evolved [23]. Due to their vascular system and megaphylls that sets them apart from primitive lycophytes, ferns represent a major step in the evolutionary sophistication of land plants. However, they lack flowers and seeds like the higher gymno- and angiosperms.

Bracken fern (*Pteridium aquilinum* (L.) Kuhn, Dennstaediaceae), the study system of the present investigation, is considered one of the world's most widespread plants and the most common fern occupying a variety of habitats [24]. Bracken is a long-lived, aggressive weed with a large rhizome system containing the plant's carbohydrate and nutrient reserves. This extensive rhizome system enables bracken to re-establish after biotic or abiotic stresses such as fire [25]. This persistent lifestyle in combination with the ability to colonize almost any habitat confers a huge ecological advantage to bracken. One important reason for this tremendous success is the fact that only few insects utilize bracken as their food source. Many of the insect species found feeding on bracken are bracken specialists including hymenopteran sawflies (*Strongylogaster sp.*, *Aneugmenus sp.*, *Tenthredo sp.*), lepidopteran larvae, dipterans like *Chirosa* sp. and *Dasyneura* species, and sucking insects such as *Macrosiphum sp.* (Aphidae) [26], [27]. Moreover, also some parasitic fungi such as the ‘curl-tip’ disease-causing *Ascophyta pterdis* and *Phoma aquilina* have been reported to infect bracken [28]. One explanation for this relatively restricted spectrum of natural enemies may be the plant's extensive array of chemical defenses, which include a diverse number of secondary compounds such as sesquiterpene indanones, cyanogenic glycosides, phytoecdysteroids and tannins [29]–[31]. Although the above-mentioned heavy armament with several direct defences is well-documented for this fern species, it is largely unknown whether primitive plants like ferns also feature the emission of VOCs as part of their defense syndrome and if so, what are the regulatory events that lead to their activation. Recently Imbiscuso et al. have reported oxidative burst and emission of volatile terpenoids after herbivory in *Pteris vittata* [9]. This fern hyper-acumulates arsenic in its fronds, which has been shown to act as defence against herbivores and pathogens. These authors could show that *P. vittata* can distinguish herbivory from mechanical damage by means of ROS (reactive oxygen species) production. Apart from this report, there is virtually no data available regarding indirect defence responses in lower plants such as ferns. In the present study, we investigate the regulatory events underlying VOC emission in the bracken fern *P. aquilinum*. Studying this trait, which functions in all higher plants studied so far as an indirect defence, in a phylogenetically more ancient plant like bracken might shed light on the evolutionary origin as well as the ancestral function of VOC emission.

In this study, we asked whether *P. aquilinum* does emit VOCs at all, and if so, whether the same signaling pathways that control VOC emission in higher plants also regulate this trait in *P. aquilinum*. These analyses were greatly aided by the wealth of information that was available on the VOC emission in higher plants [2], such as the response to certain elicitor treatments, the biosynthetic pathways and regulating molecules involved, as well as the quantitative and qualitative composition of the VOC blends emitted upon different treatments. In particular, the following questions were addressed:

- Does the bracken fern emit VOCs upon treatment with JA and other elicitors known to induce VOC production in higher plants (OPDA, α-linolenic acid, coronalon, and the pore-forming fungal peptide alamethicin)?
- Does bracken release a similar VOC blend after simple or continuous wounding and upon feeding of a generalist (*Spodoptera littoralis*) and a specialist herbivore (*Strongylogaster multifasciata*)?
- How do endogenous oxylipins levels change upon damage and herbivory?
- If terpenoids are produced, are the same biosynthetic pathways (MEP and MVA pathways) involved that are also used by higher plants?

## Materials and Methods

### Plant and insect material

Pteridium aquilinum (L.) Kuhn, Dennstaediaceae were collected from a forest about 15 km from Jena (Germany, 50°45′45.05″N and 11°40′34.85″E) and whole plants were brought to the greenhouse for further propagation. Experiments were done on plants vegetatively propagated from these in the greenhouse and grown at a temperature of 27–30°C and 45–50% humidity, under 16 h photoperiod in Klasmann clay substrate (Klasmann-Deilmann, Geeste, Germany).

The generalist herbivore, *Spodoptera littoralis* Boisd. (Lepidoptera, Noctuidae) was reared on artificial diet (500 g of ground white beans soaked overnight in 1.2 l water, 9.0 g vitamin C, 9.0 g paraben, 4.0 ml formalin and 75 g agar boiled in 1l of water. Larvae of the specialist herbivore *Strongylogaster multifasciata* (Geoffroy, 1785) (Hymenoptera, Tenthredinidae) were collected between May–June 2009 at the same field site as the plants, identified to species level [32], and maintained until use (roughly 3–5 days) on fresh fronds of *P. aquilinum*.

### Plant treatments

Plant volatile response is known to be regulated by members of the octadecanoid signaling pathway. We used α-linolenic acid, JA, COR and the pore-forming peptide alamethicin (ALA) to test for their capacity to induce VOC emission in bracken fern. Since ion fluxes and membrane depolarisation are elements of the early responses to herbivory [21], ALA was applied to evaluate the significance of pore formation for VOC-induction in bracken fern [20], [33].

For elicitor treatments, JA (1 mM), OPDA (1 mM), α-linolenic acid (2 mM) and COR (100 µM) were sprayed as aqueous solution on the fern fronds of intact plants. These concentrations were chosen based on previous literature reports, in which these compounds have been shown to induce VOC emission in other plant species [20], [33], [34]. This treatment was repeated after 30 min and plants were allowed to dry. The ALA treatment was done by placing plantlets for 24 h in ALA solution at a concentration of 10 µg ml^−1^ water (ALA, Sigma, St. Louis). ALA was initially dissolved in methanol at 10 mg ml^−1^ and this stock solution was diluted in tap water to obtain the final concentration. Fronds were damaged mechanically by puncturing 2–3 rows of holes with a pattern wheel. Continuous mechanical damage was inflicted using the MecWorm system [18] for 24 h programmed to punch 10 holes per minute and VOCs were collected simultaneously. For herbivore treatments, 2–6 larvae of *S. littoralis* or *S. multifasciata* (both reared on fern diet) were placed on each frond, allowed to feed for a day and VOCs were collected for 24 h. *S. littoralis* larvae were allowed to feed on fern fronds for 1–2 days before the onset of the experiments.

Oral secretions (OS) were collected from third instar larvae of *Spodoptera littoralis* grown on *P. aquilinum* diet, or from field-collected *Strongylogaster multifasciata* larvae. To reproducibly mimic feeding of an herbivore, 20 µl of the OS was diluted 1∶1 with de-ionized water and applied to mechanically damaged (pattern wheel) intact fronds. Mechanically wounded fronds treated with water served as a control in all experiments.

JA was prepared by saponification from commercially available MeJA (Sigma - Aldrich Chemie GmbH, Steinheim, Germany). COR, OPDA and the deuterated standards for phytohormone analysis were synthesized according to literature procedures [35], [36].

### Inhibition of VOC emission

Terpenoids are the main constituents of the VOC blend emitted from herbivore-induced plants. In higher plants, these VOCs are synthesized via either the MVA (mevalonic acid) or the MEP (methylerythritol phosphate) pathways. In order to understand the origin of the terpenoids produced by bracken fern, each of these pathways was specifically inhibited using certain inhibitors. Fosmidomycin was used to inhibit the DXP-reductoisomerase of the MEP pathway and mevinolin to block the HMGR-CoA reductase, the main enzyme of the MVA pathway [37]. For inhibitor treatments, plantlets were cut and immediately placed in 100 µM of fosmidomycin (synthesized following a patent of the Fujisawa Pharamaceutical Company, [38]) or mevinolin (Fluka Chemie GmbH, Buchs, Switzerland) solution for 24 h prior to elicitation of VOCs by JA. The lactone of mevinolin was converted into its open acid form prior use according to published protocols [39].

### Oxylipin analysis

For analysis of the octadecanoids JA, JA-Ile and OPDA, 0.2 g of frozen leaf tissues were homogenized to a fine powder in liquid nitrogen. 1.0 mL of ethylacetate spiked with 200 ng [^2^H_2_]JA and 40 ng [^13^C_6_]JA-Ile was added to the samples and after vortexing the samples were centrifuged for 15 min at 13,200 *g* (4°C). The upper organic phase was transferred into a fresh tube and the leaf material was re-extracted with 0.5 ml ethylacetate. The organic phases were pooled and evaporated to dryness. The dry residue was reconstituted in 0.4 mL of 70/30 (v/v) methanol/water for analysis with an LC-ESI-MS/MS instrument (Varian 1200 Triple-Quadrupole-LC-MS system; Varian, Palo Alto, CA). Ten µl of the sample were injected in a ProntoSIL® column (C18-ace-EPS, 50×2 mm, 5 µm, 120 Å, Bischoff, Leonberg, Germany) connected to a pre-column (C18, 4×2 mm, Phenomenex, Torrance, CA). As mobile phases 0.05% formic acid in water (solvent A) and methanol (solvent B) were used in a gradient mode with the following conditions: time/concentration (min/%) for B: 0.0/15; 2.5/15; 4.5/98; 10.5/98; 12.0/15; 15.0/15; time/flow (min/mL): 0.0/0.4; 1.5/0.2; 1.5/0.2; 10.5/0.4; 15.0/0.4. Compounds were detected in the ESI negative mode and multiple reaction monitoring (MRM) mode [40]. Quantification of endogenous OPDA was performed by external calibration with commercial OPDA (Cayman, Ann Arbor, MI) and the [^2^H_2_]JAIle.

As a next step, the precursors of JA biosynthesis namely the hydroperoxy and free fatty acids were analyzed in order to understand precursor availability. Hydroperoxy-fatty acids were extracted with the chloroform-methanol method as follows: 1 g of frozen leaf material was homogenized in 10 ml glass tubes containing 3.75 ml of ice-cold 2/1 (v/v) chloroform/methanol spiked with 5 ng of 15-hydroperoxy-eicosadienoic acid (Cayman Chemicals). After adding of 1.25 ml chloroform and 1 ml water, samples were vortexed and the phases were separated. The organic phase was collected and the water phase re-extracted with 3 ml hexane. The hexane and chloroform fractions were pooled and the solvent was evaporated under a stream of nitrogen. The samples were reconstituted in 70% (v/v) methanol/water and analyzed by liquid chromatography-(ESI)-tandem mass spectrometry (LC-MS/MS) as previously described [40]. Commercial 13*S*-(OOH)-18:3 and 9(*R/S*)-OOH-18:3 were used as standards (Cayman Chemicals).

For fatty acid analysis, 0.5 g of leaf material was ground in liquid nitrogen, transferred into 10 ml glass tubes containing 2 ml of 2-propanol and quenched by heating for 10 min at 80°C. On cooling, 3 ml of hexane and 2 ml of 6.7% (w/v) Na_2_SO_4_/water were added and after centrifugation, the organic phase was collected and the aqueous phase re-extracted with 3 ml of 3/2 (v/v) 2-propanol/hexane. The organic phases were combined, the solvent evaporated under a stream of nitrogen and the samples reconstituted in 1 ml of 1% (v/v) H_2_SO_4_/methanol. The samples were heated for 1 h at 75°C and the fatty acid methyl esters (FAMES) were extracted with hexane. FAMES were analyzed in a Varian CP-3800 GC coupled with a Varian Saturn 3800 ion trap MS in electron ionisation (EI; 70 eV) mode (Varian, Palo Alto, CA). 1 µl of the sample was injected in splitless mode on a DB-WAX column (30 m×0.25 mm I.D., 0.25 µm film thickness, Agilent, Boeblingen, Germany) with helium at a constant flow of 1.0 ml min^−1^ as the carrier gas. The injector was at 230°C. The oven temperature program was: 130°C for 5 min, 220°C at 3.0°C/min, 5°C/min ramp to 240°C and hold for 1 min. EI spectra were recorded on Scan mode from 40 to 400 amu. Quantification was performed in the linear range of detection and based on calibration curves generated with increasing concentrations of commercial FAMES (fatty acid methyl esters) mixes (Matreya, Pleasant Gap, PA) and the IS (17∶0).

### VOC collection and analysis

Treated fronds were bagged individually in a PET foil ‘*Bratschlauch*’ (Toppits®, Melitta, Minden, Germany) that does not emit detectable volatiles by itself (see e.g. [19]) and VOCs were collected in a push/pull system as decribed in [41]. VOCs emitted from each frond were collected continuously for 24 h on charcoal traps (1.5 mg charcoal, Gränicher & Quartero, Daumazam sur Azize, France) by pulling air at about 500 ml min^−1^ using a 12 V vacuum pump (Gast Manufacturing, Benton Harbor, USA). The traps were eluted with 2×20 µl dichloromethane containing 200 ng µl^−1^ of 1-bromodecane as an internal standard. Leaves were dried for dry weight determination. VOCs samples were analysed on a Thermo Finnigan Trace GC-MS (Thermo, Bremen, Germany) equipped with a fused silica Alltech EC5 column (15 m×0.25 mm internal diameter×0.25 µm film thickness) using 1.5 ml min^−1^ helium as carrier gas. Separation was achieved under programmed conditions (45°C for 2 min, 10°C min^−1^ to 200°C, then 30°C min^−1^ to 280°C for 1 min; injector temperature: 220°C). MS analysis was performed in electron impact full-scan mode at 70 eV with source temperature at 200°C and GC interface temperature at 250°C. Compounds were identified tentatively by comparison to the NIST database and subsequently collated with spectra from reference compounds (Sigma-Aldrich, Germany). Individual compounds were quantified with respect to the peak area of the internal standard and related to the dry weight of the frond.

### Statistical analysis

Differences between treatments were evaluated as a ‘general linear model’ command with ‘treatment’ as fixed and ‘plant individual’ as random factor. For multiple comparisons, LSD post hoc test tests were used in case variances were homogeneously distributed and Tamhane's T2 post hoc test if this assumption was violated. All statistical analyses were done using SPSS 17.0 (SPSS Inc., Chicago, USA).

## Results

### VOC emission from *P. aquilinum* after various treatments

**Figure 1:**
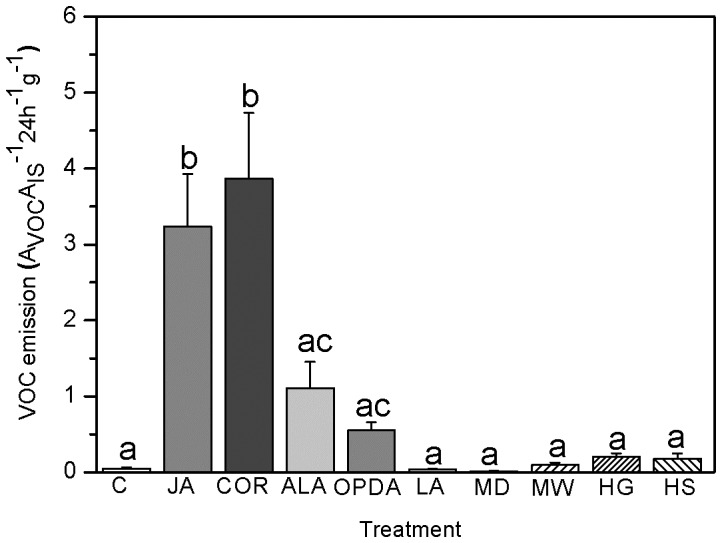
Total mean amounts (±95% confidence interval) of volatile organic compounds (VOCs) emitted from *P. aquilinum* after various treatments: C, control (tap water); JA, jasmonic acid (1 mM); COR, coronalon (100 µM); ALA, alamethicin (10 µg ml^−1^); OPDA, 12-oxophytodienoic acid (1 mM); LA - linolenic acid (2 mM); MD, simple mechanical damage by pattern wheel; MW, mechanical damage by Mecworm; HG, damage by generalist herbivore (*Spodoptera littoralis*); HS, damage by specialist herbivore (*Strongylogaster multifasciata*). The relative amounts of volatiles were determined as the ratio of peak area of a particular compound (A_VOC_) to the peak area of an internal standard (A_IS_) per gram weight of the dried frond. Different letters indicate significant difference between treatments (ANOVA: *F*_9, 39_ = 12.7, *P* = 0.01, LSD post hoc test: *P*<0.02, *n* = 3–6).

**Figure 2:**
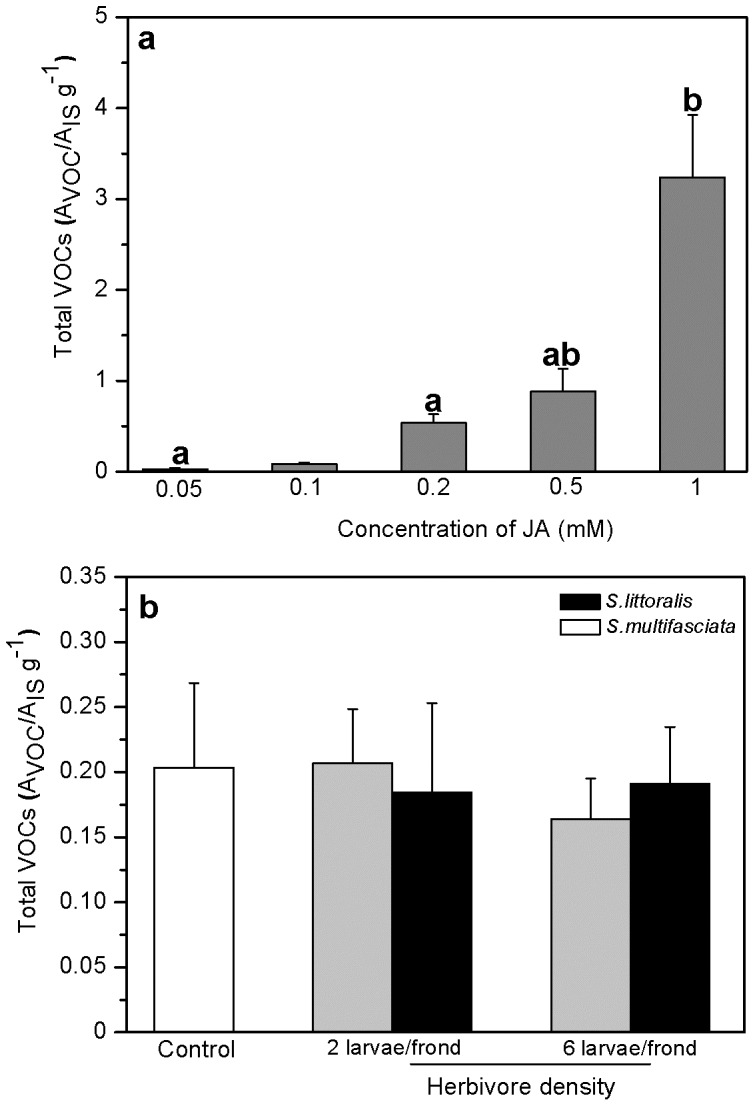
Dose-dependency of the mean total VOC emission rates (±95% confidence interval) upon a application of different JA concentrations, post hoc and b increasing herbivore density. The mean total VOC emission rates were determined as the mean of sum of all VOCs (determined as ratio of peak area of a particular compound (A_VOC_) to peak area of an internal standard (A_IS_) per gram weight of the dried frond) emitted after each treatment. Different letters indicate significant difference between the treatments (a univariate ANOVA on log-transformed data: *F*_5, 11_ = 65.5, *P* = 0.01, LSD post hoc test: *P*<0.01, *n* = 3, and b univariate ANOVA: *F*_4, 19_ = 0.7, P = 0.7, *n* = 4–6).

Interestingly, neither feeding of the specialist herbivore *Strongylogaster multifasciata* nor the generalist herbivore *Spodoptera littoralis* on intact fronds increased VOC emission rates in bracken (Fig. 1). Even increasing the number of herbivores from 2 to 6 larvae per frond did not result in enhanced volatile emission (Fig. 2b, LSD post hoc test, *P*>0.05, *n* = 4–6). Damaging the fern fronds using a pattern wheel (singular damaging event) induced low VOC emission levels that were statistically indisting-uishable from undamaged controls. To verify whether this result was merely the consequence of the damage levels, we employed a mechanical device (i.e. ‘Mecworm’ [18]) to inflict a continuous and long-lasting damage that mimics insect feeding both in terms of damage time and damaged leaf area. However, also the Mecworm treatment did not significantly increase VOC release as compared to a singular event of wounding (Fig. 1, LSD post hoc test after univariate ANOVA, *P*>0.05, *n* = 3–6).

Next, The total amount of VOCs released by bracken upon treatment with various elicitors (i.e. JA, COR, OPDA, α-linolenic acid, and ALA) was analyzed and compared to the effect of mechanical wounding and herbivore damage (Fig. 1). The total amount of VOCs emitted was generally higher in elicitor-treated plants relative to mechanically damaged and control plants (Fig. 1, LSD post hoc test after univariate ANOVA, *P*<0.02, *n*≥3–6 per treatment). Both coronalon (COR) and jasmonic acid (JA) induced the highest production levels of VOCs of all treatments.

**Figure 3:**
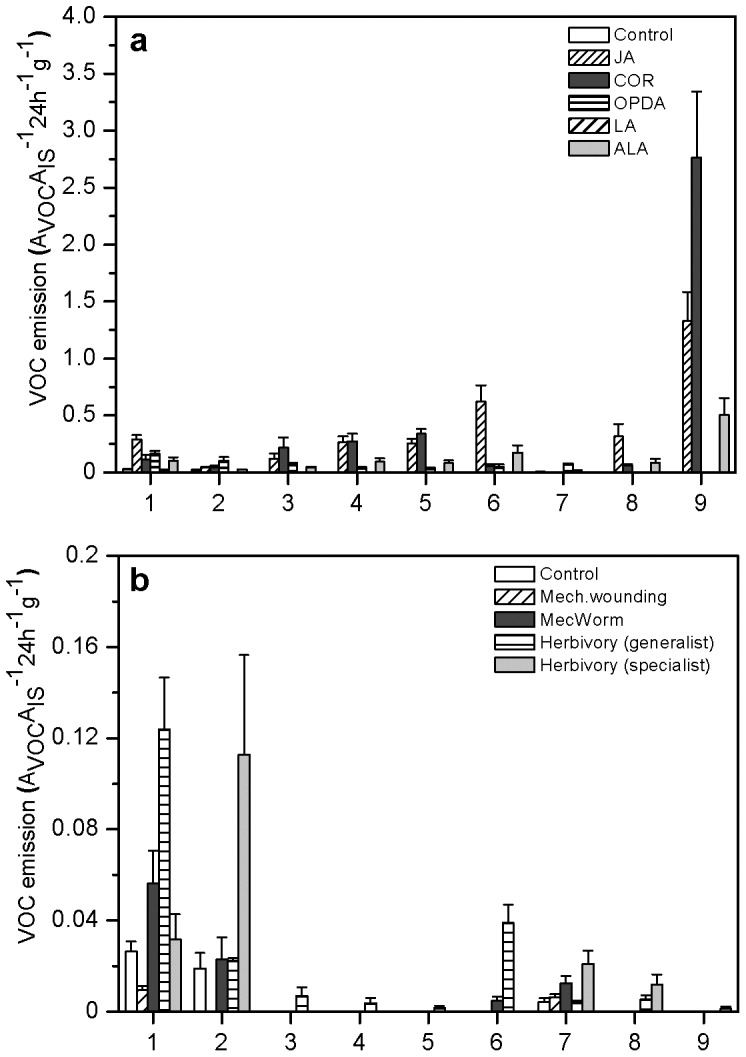
Qualitative differences in the mean VOC emission rate (±95% confidence interval) after various treatments (same as in **Fig. 1**). a Elicitor treatments (C, JA, OPDA, COR, LA and ALA) and b Damage treatments (C, MD, MW, HG and HS). The relative amounts of volatiles were determined as the ratio of peak area of a particular compound (A_VOC_) to peak area of an internal standard (A_IS_) per gram weight of the dried frond. Nine compounds were identified from the VOCs blends: 1: 1-octen-3-ol, 2:3-octanol, 3: p-cymene, 4: limonene, 5: γ-terpinene; 6: linalool; 7: nonanal; 8: α-terpineol; 9: (*E*)-β-farnesene.

The quantitative increase of VOC emission after JA treatment also resulted in strong qualitative changes relative to control plants. 17 new compounds were emitted upon JA treatment, namely, 1-octen-3-ol, 3-octanol, 2-octen-1-ol, octanone, β-pinene, p-cymene, limonene, ethylhexanol, γ-terpinene, linalool, nonanal and α-terpineol, decanal, indole, (*E*)-(β)-farnesene, aromadendrene epoxide, as well as a methylester of palmitic acid. Qualitative composition of the emitted VOC blends also differed strongly between different treatments (Fig. 3a and 3b). (*E*)-β-farnesene was the most dominant compound emitted after JA or COR treatment (Fig. 3a). 1-Octen 3-ol and 3-octanone were released after both damage- and elicitor treatments and to a small extent also from control plants (Fig. 3a and 3b). ALA induced a VOC blend that closely resembled the one induced by treatment with OPDA, which is a precursor of JA, with the exception that (*E*)-β-farnesene was only detected after ALA-, but not after OPDA treatment. Limonene was emitted after all elicitor treatments, except after α-linolenic acid- and damage treatments. Interestingly, herbivory inflicted by a generalist herbivore (*S. littoralis*) induced the emission of limonene, yet in very small amounts (Fig. 3b).

To verify to which extent the observed VOC emission pattern was dose-dependent, the JA concentration applied to each intact frond was varied and the resulting emission rate of VOCs quantified. Fronds, which have been treated with different JA concentrations ranging between 0.05 mM and 1 mM, indicated that a concentration of 1 mM JA was required to significantly increase total VOC emission levels over control levels (Fig. 2a, LSD post hoc test, *P*<0.03, *n* = 4).

In summary, bracken emitted a characteristic pattern of VOCs upon JA and other elicitor treatments, but neither mechanical damage nor damage, inflicted by natural herbivores, induced such a VOC profile.

### Oxylipin analysis

**Figure 4:**
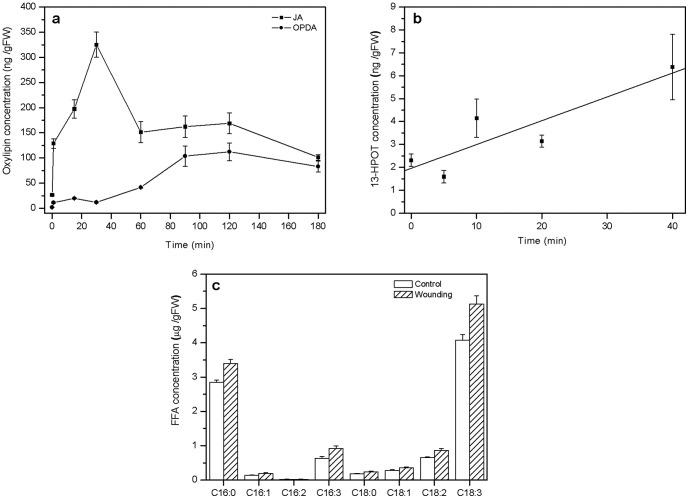
Analysis of oxylipins. **a** Quantification and time course of mean JA and OPDA levels (±95% confidence interval) after simple mechanical wounding of three individual replicates per time point. **b** Quantification and time course of mean 13-HPOT concentrations (±95% confidence interval) after mechanical wounding of four replicates per time point. **c** Mean (±95% confidence interval) content of total esterified fatty acids before and 30 min after mechanical damage. Fatty acid content of three individual replicates is indicated as µg per gram fresh weight of the tissue.

To unravel whether herbivory or tissue damage results in increased levels of endogenous JA as is known from higher plants, we monitored changes in the endogenous JA levels and its immediate precursors after both treatments. Initially, a kinetic study was conducted by measuring changes in the levels of the phytohormone JA, and its precursor 12-oxophytodienoic acid (OPDA) upon wounding as a function of time (Fig. 4a). Endogenous JA levels of the fronds reached a maximum after 30 minutes of wounding, while no major burst in OPDA levels was detected (Fig. 4a). Likewise, no major burst in the accumulation of the JA precursor 13-hydroperoxy-linolenic acid (13-HPOT) could be detected within 40 min after wounding (Fig. 4b). We also analyzed the total amounts of esterified fatty acids in bracken before and 30 min after wounding and determined that total fatty acids accumulated to about 8.8±0.35 (mean ± 95% confidence interval) µmol g fresh weight^−1^, of which saturated fatty acids (16∶0 and 18∶0) accounted for 34% and unsaturated fatty acids (16∶1^Δ7^,16∶2^Δ7,12^,16∶3^Δ7,10,13^,18∶1^Δ9^,18∶2^Δ9,12^,18∶3^Δ9,12,15^) constituted 65% of the total amount of these molecules (Fig. 4c). Mechanical damage did not significantly change the amounts of total fatty acids in leaves within 30 min after the stimulus, including the levels of α-linolenic acid (18∶3^Δ9,12,15^), the initial precursor for JA biosynthesis (Fig. 4c).

**Figure 5:**
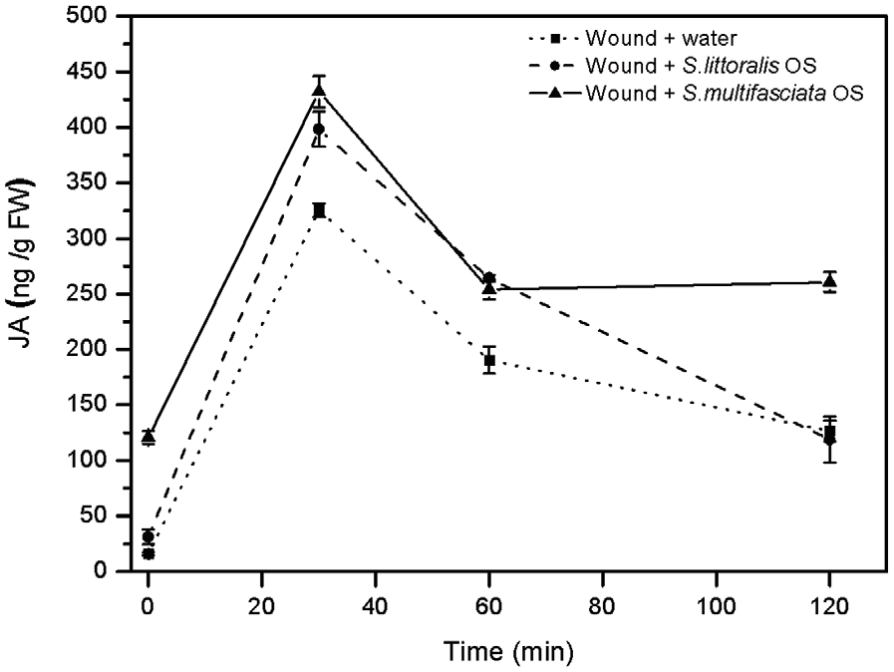
Quantification and time course of mean endogenous JA levels (±95% confidence interval) after wounding and treatment. JA levels were quantified at different time points after mechanical wounding (Wounding + water), wounding + application of oral secretion (OS) collected from *S. littoralis*, and wounding + OS from *S. multifasciata*, both herbivores reared on fern diet. Data represents five individual replicates per time point and treatment.

To analyze the effect of herbivory on endogenous JA levels, oral secretions (OS) from generalist herbivores (*Spodoptera littoralis*; reared on fern diet; Fig. 5) and specialist herbivores (*Strongylogaster multifasciata*; reared on fern diet; Fig. 5) were applied to mechanically damaged fronds. Interestingly, JA levels did not increase significantly relative to wounded tissue before and after generalist or specialist OS application (Fig. 5, Tamhane's T2 post hoc test: *P*>0.05, *n* = 5).

### VOC emission after inhibitor treatment

**Figure 6:**
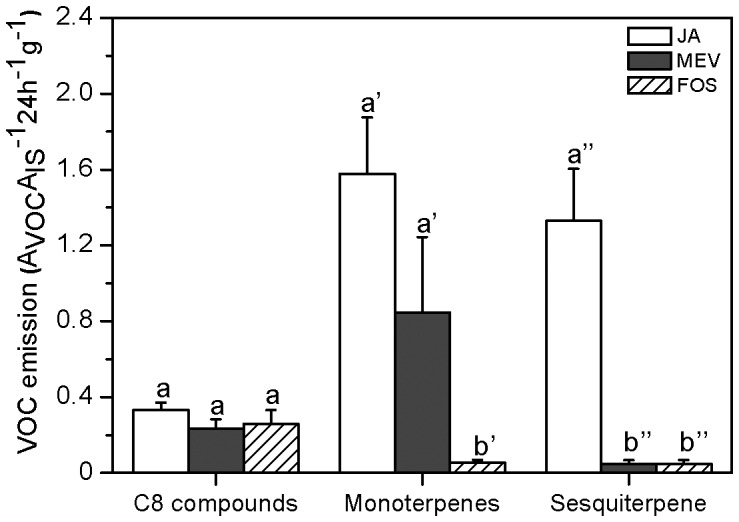
Mean VOC emission rate (±95% confidence interval) upon application of the inhibitors fosmidomycin (FOS) and mevinolin (MEV) prior to JA treatment. VOCs emitted in response to treatments are grouped according to their chemical class (see Fig. 3a and 3b, C8 compounds (compounds 1–2; univariate ANOVA: *F*_2, 13_ = 6.6, *P* = 0.01, LSD post hoc test *P*<0.02; monoterpenes (compounds 3–7; univariate ANOVA: *F*_2, 13_ = 9.37, *P* = 0.03, LSD post hoc test *P*<0.03), and sesquiterpene (compound 9; univariate ANOVA on log-transformed data: *F*_2, 13_ = 10.7, *P* = 0.003, LSD post hoc test, P<0.05). Different letters indicate significant difference between the treatments.

Since bracken produced both mono- and sesquiterpenes in response to JA treatment, we investigated which metabolic pathways were involved in the production of these compounds using specific inhibitors (fosmidomycin and mevinolin). Treatment with these inhibitors did not affect the emission rates of C_8_ volatiles, namely 1-octen-3-ol and 3-octanol (Fig. 6, LSD post hoc test after univariate ANOVA, *P*>0.05, *n* = 5), compared to JA-treated plants, whereas the amount of monoterpenes emitted after inhibition with fosmidomycin was significantly lower compared to JA-treated plants (LSD post hoc test after univariate ANOVA, *P*<0.02, *n* = 5). The observation that fosmidomycin was more effective in blocking monoterpene production than mevinolin, indicated that the MEP pathway accounted for the formation of these compounds (Fig. 6). On the other hand, emission of (*E*)-β-farnesene was suppressed by both inhibitors by almost 90% as compared to JA treatment, which suggests that the plastid-derived MEP pathway supports the formation of both mono- and sesquiterpene synthesis in bracken fern.

## Discussion

Several fascinating functions for the emission of plant VOCs have been discovered. However, the basic questions why there is such a structural diversity of VOCs as well as how these evolved to function as an indirect defence mechanism in plants remain largely unanswered. VOC emission could have originated as a direct defence strategy against herbivores or pathogens or as a means to attract pollinators [42]. Although it is difficult to reconstruct the evolutionary origin of VOC emission, comparative studies on phylogenetically ancient plant species can provide insight into the ancestral origin and function of VOC emission.

For many higher plants, it is well known that insect feeding induces the emission of a VOC blend that strongly resembles the one released upon exogenous application of JA [1], [5], 16], [19]. The aim of this study was to investigate whether i) the emission of VOCs in the evolutionary ancient fern *P. aquilinum* is also regulated by jasmonates, ii) the same elicitors (chemical elicitors, herbivory, and mechanical damage) that are known from higher plants trigger VOC emission in bracken. The impact of mechanical damage as a component of herbivore feeding was studied using a computer-controlled device (i.e. ‘Mecworm’).

When ferns were treated with COR, the VOC blend emitted resembled the one emitted from JA treated plants both qualitatively and quantitatively (Figs. 1 and 2a). This observation differs from the responses known from higher plants, where the elicitation with COR induces a much more complex spectrum of VOCs than JA [35]. Furthermore, in *Phaseolus lunatus*, treatment with ALA mainly induced the emission of homoterpenes; the emitted VOC blend resembled OPDA-treated, rather than JA-treated plants. In the present study, ALA treatment elicited a VOC profile that was more similar to OPDA-treated than JA-treated plants [20]. The major compound observed upon JA- and COR-treatment was (*E*)-β-farnesene (Fig. 3a), a sesquiterpene that has been previously described to function as an aphid alarm pheromone [43], [44]. Interestingly, (*E*)-β-farnesene was also the most dominant VOC emitted from the fern *Pteris vittata* [9]. Previous studies have shown an increased emission of this compound in response to simple mechanical damage [45], herbivory [4], [6], and JA treatment [46], [47]. Furthermore, it is interesting to note that bracken emits 1-octen-3-ol, a volatile typical for mold fungi [48], mosses [49], beans [50], and mint plants [51]. In mushrooms, α-linoleic acid is the precursor for the biosynthesis of 1-octen-3-ol, which proceeds via the LOX/HPL mediated pathway. Recently, it has been shown that this compound can induce defence genes in *Arabidopsis thaliana* [52]: Treatment with 1-octen-3-ol increased the resistance of the plant against the pathogen *Botrytis cinerea*, suggesting that 1-octen-3-ol may be recognized by the plant as a signal for presence of fungal pathogens [52].

In higher plants, terpenoids are synthesized via the MEP or MVA pathways and studies using inhibitors of either pathways to dissect the origin of the mono- or sesquiterpenes have shown, that there exists a crosstalk between the two pathways [53]. For example, in lima bean, herbivory stimulated the emission of the homoterpene DMNT (4,8-dimethylnona-1,3,7-triene) through the cytosolic MVA pathway [37]. When the latter pathway was blocked, this deficiency was compensated by channelling the biosynthesis of DMNT via the MEP pathway [37]. Evolutionary studies indicate that each domain of life has evolved a characteristic isoprenoid biosynthesis pathway. While the MVA pathway is considered to be the major route to isoprenoids in eukaryotes, archea as well as in bacteria, the MEP pathway is common to bacteria and photosynthetic eukaryotes [54]. Plants possess both the MVA pathway and acquired the MEP pathway by horizontal gene transfer from endosymbiotic cyanobacteria, the ancestor of plastids. In the ancient bracken fern, fosmidomycin effectively blocked both mono- and sesquiterpene emission while mevinolin effectively inhibited sesquiterpene biosynthesis, indicating a signalling crossstalk and a non-independence of these two pathways. Taken together, in the fern species studied here (*Pteridium aquilinum*), elicitors like JA, COR OPDA, α-linolenic acid, and ALA induced the emission of certain VOCs, which is in line with previous studies using higher plants. Interestingly, continuous damage by the ‘Mecworm’ did not result in significantly increased VOC emission levels in the fern (Fig. 1).

The observation that neither mechanical damage (Fig. 1) nor herbivory could significantly induce VOC emission, is in contrast to what is known from higher plants. A possible ecological explanation for this observation could be that bracken fern depends more on direct than indirect defences to protect itself from herbivore feeding [55]. Indeed, bracken is known to be highly toxic and generally unattractive to insects or mammalian herbivores [29], [56]. Among the few insects feeding on bracken, a predominance of sawflies has been reported [23], [57]. Consistent with these reports, *S. multifasciata*, which was used for our experiments, was observed to be an herbivore of bracken at its natural growing site (RV, personal observation). Larval feeding of this herbivore, however, resulted in much lower emission levels of VOCs than were released from elicitor-treated ferns (Fig. 3b). Also, increasing the larval density per frond did not result in higher VOC emission levels (Fig. 2b), indicating that the lack of an inductive effect was not due to too low damage levels applied. Finally, also increasing the time of volatile collection after herbivory by both generalist and specialist insects from 24 h to 3 days did not reveal significantly increased VOC emission levels (results not shown).

Endogenous levels of JA in response to mechanical damage and herbivory did not differ from each other and did not exceed 500 ng (g FW)^−1^ (Figs. 5 and 6). These observations together with the finding that exogenous JA application could trigger VOC emission while herbivory or mechanical damage could not, implies that the lack of VOC emission after herbivory could be due to too low internal JA levels. Probably, the endogenous JA level did not exceed a certain ‘threshold’ value required for the biosynthesis and release of VOCs, which, however, could be attained by external application of the phytohormone. Indeed, a positive correlation between insect-induced internal JA levels and the resulting volatile emission rate was reported in corn - *S. exigua* interactions [47], which corroborates our interpretation that exceeding a threshold value of JA might be required to initiate VOC production in bracken. This is further supported by the results of our experiments in which increasing JA concentrations were applied to bracken plants. Here, only a concentration of 1 mM of JA could induce significantly increased emission rates of VOCs. Moreover, our observation that increasing the number of herbivores or the days of feeding did not significantly alter VOC emission is in line with the interpretation that bracken does probably not rely on VOCs as an inducible defence strategy. Whether the lacking response in terms of VOC emission of bracken after herbivory is really due to this endogenous ‘threshold’ level of JA requires further investigation.

Interestingly, *Ginkgo biloba* has been shown to produce increased amounts of VOCs upon JA treatment, but failed to emit any volatiles after tissue damage [58]. This observation resembles our findings in bracken, thereby indicating that the biosynthetic machinery to produce VOCs is also present in lower plant species. Which biotic or abiotic factors can activate these responses, however, remains elusive in both cases. A possible hint is provided by the observations that both COR and ALA induced VOC emission in bracken. COR is a structural mimic of the ultimate signalling molecule jasmonoyl-isoleucine (JA-Ile). The strong response to COR demonstrates that in ferns probably a protein complex, similar to the JAZ-proteins in higher plants [14], [15] is responsible for binding the jasmonate-linked signal. Moreover, ALA is active since the peptide forms a hexameric pore [59] in biomembranes, which allows ions to cross the membrane thereby starting defense responses including phytohormone production [33]. Currently, virtually all knowledge that is available on indirect defence mechanisms such as VOC emission, stems from higher plant species such as cotton, *A. thaliana*, tobacco, tomato, soybean, lima bean and maize [2], [7], [60] and our study is a first step in understanding this indirect defence mechanism in bracken fern. In light of the abovementioned findings in bracken, it will be very interesting to also investigate VOC emission in other, more ancient plant species such as conifers or gnetales. Especially, since the fern *Pteris vittata* responded with an emission of terpenoids after herbivory [9], a more detailed knowledge of VOC emission in these plant species in combination with phylogenetic data will help to trace back the point in evolutionary time at which the plant-internal recognition mechanisms for herbivore damage and the downstream octadecanoid signalling pathway were linked to the VOC producing machinery.
